# Transmediastinal Gunshot Wound in a Pregnant Patient with Stable Hemodynamics

**DOI:** 10.1055/s-0040-1715142

**Published:** 2021-01-19

**Authors:** Ozhan Ozdemır, Cemal Resat Atalay

**Affiliations:** 1 Department of Obstetrics and Gynecology, Gulhane School of Medicine, University of Health Sciences, Ankara, Turkey; 2 Department of Obstetrics and Gynecology, University of Health Sciences, Ministry of Health Ankara City Hospital, Ankara, Turkey

**Keywords:** gunshot, transmediastinal, trauma, pregnancy

## Abstract

Transmediastinal gunshot wounds (TGWs) may lead to life-threatening injuries of vital organs such as large vessels, the esophagus, and lungs. Although they are not commonly encountered in pregnant women, additional caution should be given to these patients. Physical examination for the diagnosis and the choice of treatment modality contain controversial points in hemodynamically stable patients, and resuscitation has excessive importance due to physiological changes in pregnancy. We present a hemodynamically stable 26-week pregnant woman brought to the emergency department for TGW. She had a 1-cm diameter of bullet entrance hole on the right anterior 4 ^th^ intercostal space, 2 cm lateral to the sternum, and a 3-cm diameter exit hole on the right posterior 12 ^th^ intercostal space on the midscapular line. With our conservative approach, she had an uncomplicated pregnancy period, and gave birth to a healthy baby at term.

## Introduction

Although the exact incidence of trauma during pregnancy is not known, approximately every 1 pregnancy out of 12 is complicated with trauma, and it is one of the leading reasons for nonobstetric maternal death. 1 Approximately 9% of all traumas during pregnancy is penetrant, and handguns, knives, and shotguns are the cause in 73%, 23%, and 4% of them, respectively. 2 Maternal mortality due to penetrant traumas during pregnancy is between 3.9 and 7%, whereas fetal mortality is ∼ 73%. 2

Mediastinum is restricted with the sternum anteriorly, the spine posteriorly, and the diaphragm inferiorly, and contains vital organs such as the heart, the esophagus, large vessels, and the tracheobronchial tree. Hence, the mediastinum should be evaluated carefully and systematically in patients with transmediastinal gunshot wounds (TGWs). In patients with gunshot wounds, the prediction of the direction of the bullet inside is difficult, and the entrance hole does not give an adequate idea about the location and extent of the wound. Furthermore, the physiological and anatomical alterations during pregnancy require additional care for the evaluation and treatment of these patients. 3 Herein, we report a hemodynamically stable 26-week pregnant woman with TGW in whom the pregnancy terminated uncomplicated with conservative approach.

## Case Report

**Fig. 1:**
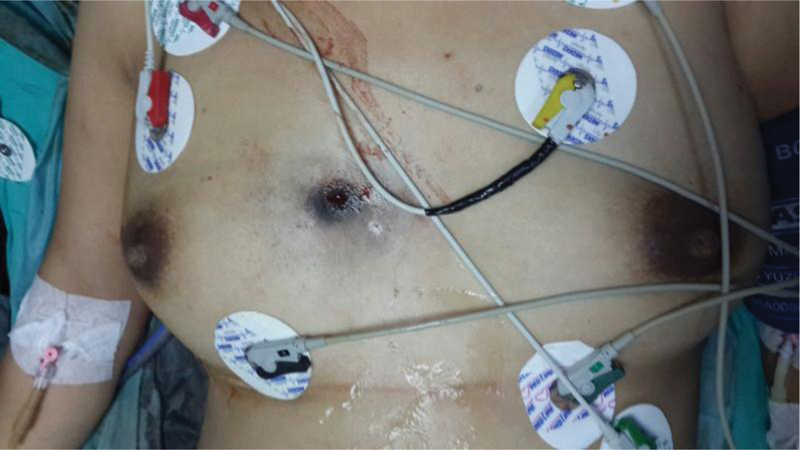
The entrance hole of the bullet with ∼1 cm diameter on the right anterior 4th intercostal space, 2 cm lateral to the sternum.

**Fig. 2:**
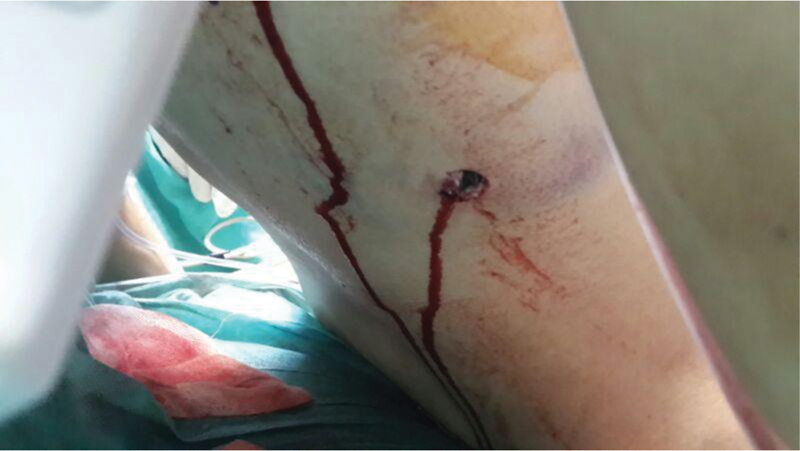
The exit hole of the bullet with ∼3 cm diameter on the right posterior 12th intercostal space on the midscapular line.

**Fig. 3:**
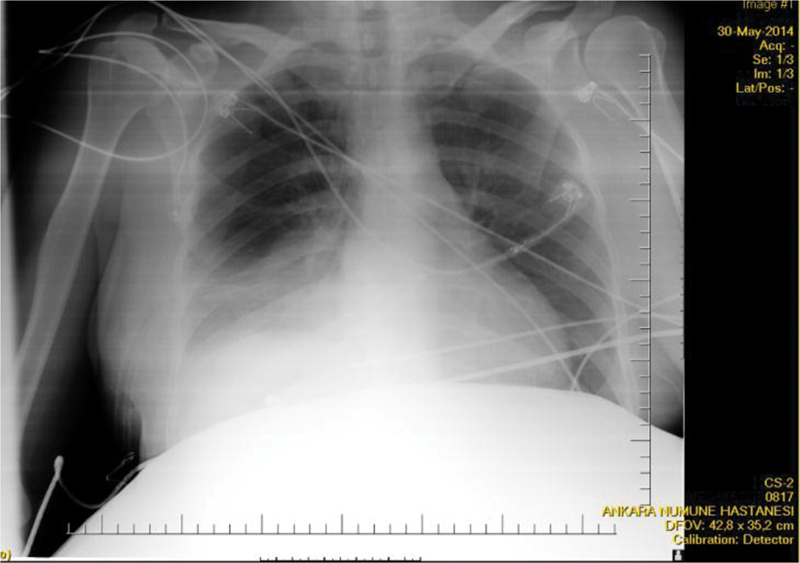
Chest radiogram following tube thoracostomy.

A 37-year-old, primiparous 26-week pregnant woman was brought to the emergency department with a gunshot wound. She had a 1-cm bullet entrance hole on the right anterior 4 ^th^ intercostal space, 2 cm lateral to the sternum ( Fig. 1 ), and a 3-cm exit hole on the right posterior 12 ^th^ intercostal space on the midscapular line ( Fig. 2 ). She was brought to the hospital in 30 minutes following the gunshot, and she was conscious, oriented and cooperated when she arrived. She was hemodynamically stable with arterial blood pressure 110/70 mm Hg, 89 pulses/minute, body temperature 36.7°C, respiration count 28/minute, and oxygen saturation 92%. Thoracentesis was performed from the right midaxillary 6 ^th^ intercostal space as no respiratory sound was heard on the right lung base, and defibrinated hemorrhagic fluid was aspirated. Following tube thoracostomy, 700 mL of defibrinated hemorrhagic fluid was acutely drained. The chest radiograph after tube thoracostomy revealed open bilateral sinuses and expanded parenchyma; however, increased density was present in right lower zones probably due to contusion ( Fig. 3 ). The echocardiography revealed normal cardiac functions with no pericardial fluid. Similarly, free fluid was not seen in the abdominal ultrasonography, and no evidence of laceration of organs or large vessels was present. In obstetric ultrasonography, single fetus matching 26 weeks with positive fetal cardiac activity, normal amniotic fluid index, and no placental pathology was seen. The patient did not have any complaints about vaginal bleeding or amniotic fluid loss, and cervical dilation and effacement was not observed in the pelvic examination. Antibiotics and steroid for fetal lung maturation were administered, and an additional 100 mL defibrinated hemorrhagic fluid was drained from the thoracostomy tube in the 1 ^st^ hour. The patient was followed with stable vital signs and hemodynamics. As the drainage from the thoracostomy tube decreased in the follow-up, the tube was exerted on the 5 ^th^ day, and the entrance and exit holes were sutured after debridement. The patient was discharged from the hospital on the 7 ^th^ day of her arrival. No obstetric complication was observed during her follow-up, and she gave birth to a healthy, 3.700 g-weighing child in the 39 ^th^ week by elective cesarean section due to cormical presentation.

## Discussion

Gunshot wounds are the second most common traumas following traffic accidents in the general population, and penetrant wounds are extremely rare in pregnant women. 4 As treatment is urgent in hemodynamically unstable patients with TGW, diagnostic tests are often emitted. In hemodynamically stable patients, imaging techniques such as ultrasonography, computed tomography (CT), esophagography or esophagoscopy, angiography, or bronchoscopy should be performed when indicated, to determine the exact location of the injury. 5

Several patients with TGW succumb to death owing to cardiac tamponade or acute fatal hemorrhage before they are brought to the hospital. Approximately 43% of the patients are hemodynamically unstable when they arrive at the hospital and need urgent surgery. The remaining 57% of patients are hemodynamically stable, and between 35 and 60% of them need urgent surgery. 6 The mortality rate of patients with TGW brought to hospital is 27%, and the majority of them are hemodynamically unstable. The mortality rate of hemodynamically stable patients is between 0 and 10%. 6 However, according to some authors, more than half of the patients with TGW are hemodynamically stable, and more than between 60 and 70% of these patients do not need surgery, and are treated with conservative approach. 7

The approach to penetrant injury and resuscitation in pregnancy is of greater importance owing to the presence of the fetus and physiological and anatomical gestational alterations. The management of penetrant injury in pregnancy requires a multidisciplinary approach comprising the anesthesiologist, obstetrician, neonatologist and trauma surgeon. Moreover, it should be individualized according to the entrance site of the wound and time of pregnancy. 8 The primary aim in the management of trauma in a pregnant woman is to obtain maternal stabilization, and the early and aggressive resuscitation of the pregnant woman is directly associated with fetal results. 9 In addition, maternal physiological and anatomical alterations should be taken into account during resuscitation. For instance, a preservative environment is formed through physiological hemodilution and hypervolemia during pregnancy, and maternal shock symptoms may not be observed until 40% of the maternal blood volume is lost. Furthermore, maternal hemodynamic measurements may not be able to correctly point out the state of uteroplacental circulation. Besides, as the buffering capacity also decreases in pregnancy, tendency to metabolic acidosis may occur in case of hypoperfusion and hypoxia. 9 The functional residual capacity of the lungs decreases during pregnancy due to elevation of the diaphragm ∼ 4 cm and increase in chest diameter ∼ 2 cm, leading to an increase in tendency to hypoxia. These alterations should be considered during interventions such as thoracostomy. If necessary, a thoracostomy tube should be placed through the intercostal space 1 or 2 above the classical 5 ^th^ intercostal space to prevent abdominal insertion. 8

Essential radiologic imaging techniques should promptly be performed if clinically indicated in traumas during pregnancy, and in this situation fetal effects may be ignored. The gold standard diagnostic method for TGW in hemodynamically stable patients is CT angiography. 10 Fetal radiation exposure of CT is > 3.5 rad (0.035 Gy), and its potential benefit should be considered in life-threatening injuries. More importantly, fetal exposures < 5 rad (0.05 Gy) do not lead to any increase in the risk of fetal anomaly, pregnancy loss or growth retardation. 1 In nonsensitized Rh negative pregnant women with major injuries, the Kleihauer-Betke test should be performed to calculate the total Rh immunoglobulin dose for prophylaxis. 11

Burack et al 10 evaluated 207 patients with mediastinal penetrant injury in the emergency department, and reported that 35 and 65% of these patients were hemodynamically unstable and stable, respectively. Twenty-six percent of hemodynamically unstable patients succumbed to death during medical interventions. The remaining 53 patients were submitted to urgent surgery and, among these, 32 patients survived. The CT angiography was normal in 80% of hemodynamically stable patients, and conservative treatment was preferred. In another study, > 60% of the patients with transmediastinal injury were hemodynamically stable and were treated conservatively. 12 Both these studies show that the conservative approach is adequate for hemodynamically stable patients with transmediastinal injury. However, several patients who are stable at first will need surgery. The decision for surgery is made by the results of imaging studies and the amount of the fluid draining from the thoracostomy tube. In our case, conservative treatment was performed, and the pregnancy terminated successfully.

## Conclusion

As a result, hemodynamically stable patients with TGW should be evaluated for their need for adequate diagnostic methods to determine the site and severity of the injury. In patients who are chosen to be treated conservatively, the anatomical and physiological alterations during pregnancy should be considered.
